# Prospective study and new concepts based on scoliosis detorsion of the first 225 early in-brace radiological results with the new Lyon brace: ARTbrace

**DOI:** 10.1186/1748-7161-9-19

**Published:** 2014-11-19

**Authors:** Jean Claude de Mauroy, Cyril Lecante, Frédéric Barral, Sophie Pourret

**Affiliations:** Clinique du Parc, Lyon, France; Orten, Lyon, France

**Keywords:** Scoliosis, Brace, Lyon brace, Concepts, Torsion, Moulding, Cad/Cam, Early results, In-brace correction

## Abstract

**Background:**

The symmetrical Lyon brace is a brace, usually used to maintain correction after a plaster cast reduction in the Cotrel’s EDF (Elongation-Derotation-Flexion) frame. The new Lyon brace or ARTbrace is an immediate corrective brace based on some of the principles of the plaster cast which are improved due to advances in CAD/CAM technology. The aim of this paper is to describe concepts of this new brace to be not only a replacement of the plaster cast, but also a definitive brace.

**Methods:**

Instead of a plaster cast, three segmental CAD/CAM moulds are made with the instantaneous full 3D raster stereography digitizer (Orten):

1. In self axial elongation
2. In shift and lumbar lordosis
3. In shift and thoracic kyphosis

A specific software (OrtenShape) makes up the overlay of the three moulds. Mould 1 is used for the pelvis and the shoulders mould 2 for the lumbar segment and mould 3 for the thoracic segment.

The mathematical basis of the ARTbrace is the torso column which is a circled helicoid with horizontal circle generator. A torso column is reproduced in the opposite direction of the scoliosis.

Like the plaster cast, the ARTbrace is worn for a “total time” of 24 hours 7 days a week without modifying the standard protocol of the Lyon brace reduction.

The prospective controlled cohort observational study of the 225 first patients treated since May 2013 is reported below.

**Results:**

The in-brace immediate reduction is: 0.7, i.e. 40% better with the ARTbrace than with a plaster cast. The correction of flat back is 9° (from 18°.4 to 28°.5 kyphosis Cobb angle). The improved aesthetic appearance is equal for rib hump and ATR.

**Conclusion:**

This first paper is an introduction with very short results and does not prejudge the final outcome. The ARTbrace can be used not only to replace the plaster cast, but also as a definitive brace. The new segmental moulding with final detorsion is even more efficient and to this day the ARTbrace is the most effective to reduce the Cobb angle of scoliosis.

**Electronic supplementary material:**

The online version of this article (doi:10.1186/1748-7161-9-19) contains supplementary material, which is available to authorized users.

## Background

The randomized control trial BRAIST study conducted by Weinstein showed that bracing is significatively effective in reducing the progression of AIS [1].

The effectiveness of a brace depends not only on the immediate in-brace reduction, many other factors are involved:

How to get the three-dimensional correction and its reproducibility.

The patient’s adherence which depends on aesthetics and tolerance [2].

Lyon management has proven its effectiveness and is not affected by the ARTbrace. The physiotherapy undertaken is identical to the classical Lyon brace [3].

The 14 recommendations constituting the SOSORT Criteria derive largely from the experience of major European centres for scoliosis treatment, like the ‘Centre des Massues’ in Lyon. They are the subject of a consensus [4]. Although the plaster cast has proven its effectiveness [5], there is no consensus on the reduction by plaster cast before bracing, which continues to be used for infantile scoliosis [6, 7], but was gradually abandoned for AIS.

## History

**Figure 1:**
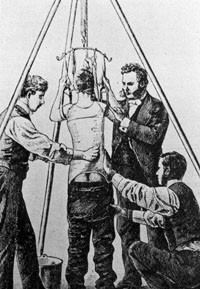
**Reduction of a scoliosis by Lewis Albert Sayre.**

In the United States, in the early twentieth century, Sayre [8] was the first to make a plaster cast in a standing posture using the mechanical principle of elongation and derotation like a spring (Figure 1).

The first modern brace was the Milwaukee brace created in 1940 by Blount based on axial elongation between the pelvis and the cervical collar.

**Figure 2:**
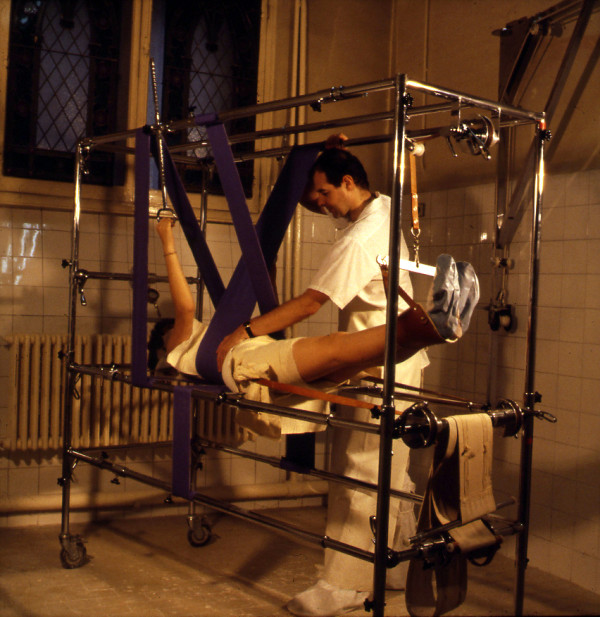
**EDF Cotrel’s frame for 3D scoliosis correction in supine position.**

In France, the Lyon brace, created in 1947 by Pierre Stagnara, was a 3D adjustable contention brace used after a plaster cast. Cotrel added a fundamental component: the flexion in the frontal plane [9]. He created a framework for three-dimensional scoliosis correction in the supine position with spine untwisting (Figure 2).

The plaster cast of the Lyon method combines several mechanical actions:

1. Supine position
2. Axial Elongation, as with the Milwaukee brace
3. In the frontal plane a 3 points action, with push and counter-pushes
4. In the sagittal plane, kyphosis is obtained by virtue of the “hammock” effect and posture of the upper limbs
5. In the horizontal plane, derotation between the pelvis and shoulders is obtained by positioning the upper fixation of the tapes at the top of the frame.

At the end of plaster cast weaning, the plaster mould to build the Lyon brace reproduces the correction obtained [10, 11].

**Figure 3:**
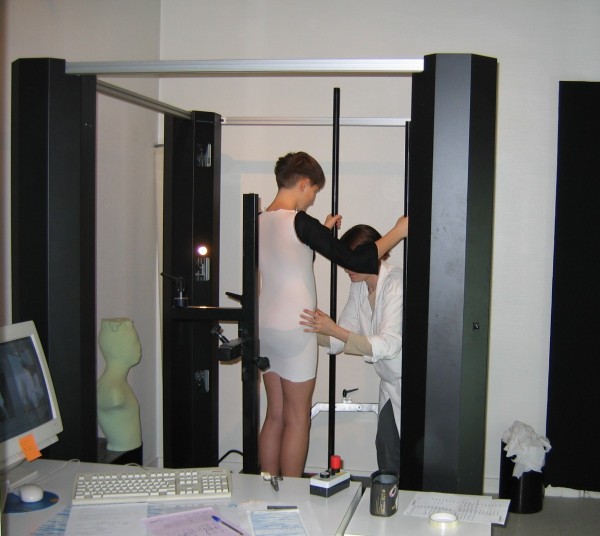
**Full 3D instantaneous raster stereography Orten.**

Since 1987, we have developed the CAD/CAM moulding whose most sophisticated version is the instantaneous full 3D raster stereography digitizer Orten [12] (Figure 3).

This surface topography device dedicated to brace moulding is able to test the modelling effect, by comparing files obtained before and after plaster cast [13]. The efficiency in brace correction between the traditional plaster mould and the CAD/CAM moulding is not obvious. A better angular in-brace correction was noted with CAD/CAM, but it was not statistically significant [14]. For Wong the in-brace reduction with the manual method is 0.321 versus 0.419 for the CAD/CAM method, which is non-significant [15]. Sankar reaches the same conclusion, but notes that patients prefer the CAD/CAM mould [16].

Many previous studies are supporting the positive results with the casting and Lyon braces [10, 11] but the difficulty and cost of making the plaster cast can also explain the challenge that prompted a need for improvements, which ultimately resulted in the development of this new design concept.

In 2013, the latest generation software (OrthenShape) allowed the overlay of different CAD/CAM moulds. The aim was to use this new software to replace the plaster cast with a new Lyon brace: the ARTbrace.

Since May 2013, all patients of JCdM were treated with the ARTbrace instead of a plaster cast. The corrective concepts and early in-brace results will be reported in this first article.

## Methods

### Study design

This is a prospective controlled cohort observational study.

The experimental hypothesis predicted that patients treated with the ARTbrace would report a significant in-brace correction of major, minor, thoracic and lumbar curves for both the main prospective group and SRS & SOSORT restrictive criteria [17–19]. Although it is difficult to compare the different braces used around the world, we present the results in the same form as the Rigo System Cheneau (RSC) results [20].

### Setting of the study: the five innovative concepts

Like (RSC) the general correction principle is detorsion and sagittal normalisation, i.e. with a minimum of distraction which usually favours the flat back [21]. However, the methodology of the ARTbrace achievement differs radically.

**Figure 4:**
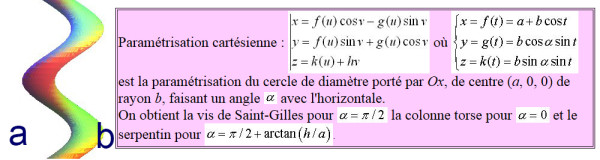
**Circled Helicoid (a) and its mathematical basis (b).**

**Figure 5:**
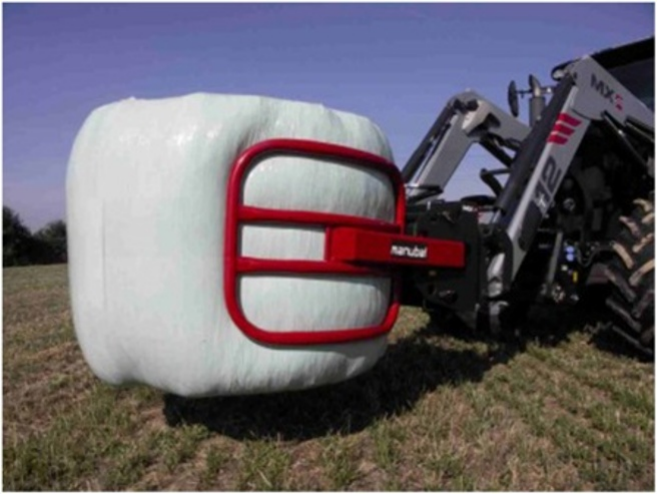
**Squeeze attachment for cylindric bales principle.**

**Figure 6:**
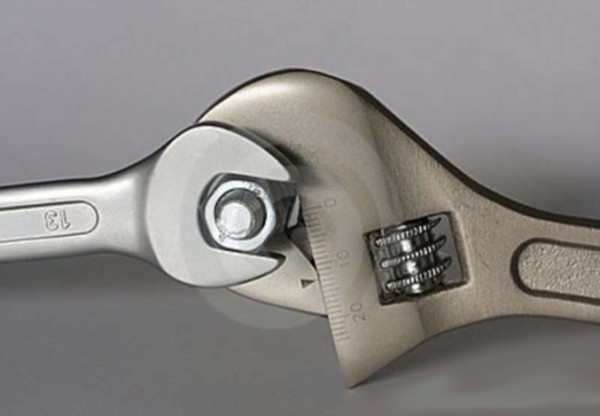
**The wrench and bolt principle.**

**Figure 7:**
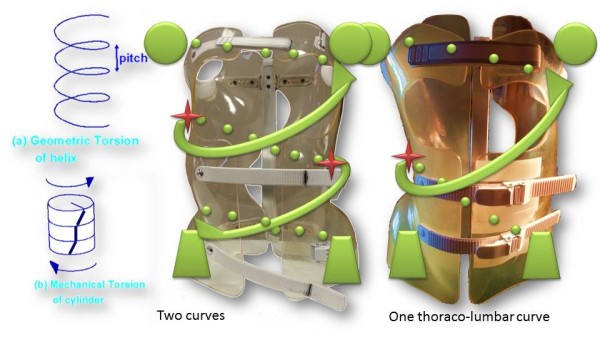
**Theoretical detorsion of the ARTbrace for one single curve and two curves.** with both **(a)** geometrical and **(b)** mechanical detorsion.

1. The mathematical basis of the torso column is the circled helicoid with horizontal generating circle described by the French mathematician Robert Ferréol [22].For a circled helicoid, the Cartesian parameterization is the parameterization of the circle with diameter carried by Ox, with center (a,0,0), with radius b, forming an angle alpha with the horizontal. For torso column alpha = 0 (Figure 4).
  The aim is to get not only a straight spine, but a reverse torso moulding opposite to scoliosis i.e. overcorrection of the scoliosis curvature. This overcorrection is possible only if the vertebral bodies are not distorted. Otherwise, we favour the correction accentuating the asymmetry of pressure on the vertebral body.
2. The second concept is that of the squeeze attachment for cylindrical hay bales. Pressures are spread over the entire cylinder surface; this is contrary to the principle of the push and counter-push of the historical Lyon brace or other three point braces. As usual in the correction of 3D deformities of the scoliotic spine, room should be provided for migration of lateral curvature, rotated vertebrae and breathing exercises. In this design, actually various 3-point pressure systems are provided to correct the lateral curvature and vertebral rotation from different anatomical planes. In the ARTbrace the shape of the brace is not a straight spine like the Sforzesco or the old Lyon brace, but an overcorrected spine with reverse scoliosis (concept 1). This is possible thanks to the superposition of two corrective bending mouldings (Figure 5).
3. The third concept is the wrench and bolt principle to “unscrew or untwist” scoliosis. For instance, the Chêneau brace uses the principle of pressure and expansion in many precise areas [23]. For a double major curve in the ARTbrace, the thoracolumbar area is the fixed point with unscrewing between this fixed point and the pelvis for lumbar curvature and the shoulder girdle for thoracic curvature. For a thoraco-lumbar curve, the fixed points are at the cranial and caudal parts of the spine and the unscrewing is done at thoracolumbar level. The pelvis is the «bolt head» which is stabilized by a symmetrical pelvic base like a key. Lumbar and thoracic segments above act as a wrench for the detorsion of scoliosis (Figure 6).
4. The fourth concept is detorsion with a fixed sagittal plane. Axial elongation brings the vertebral bodies near the central axis in the frontal plane, and by untwisting the scoliotic spine between the pelvis and the shoulder the horizontal plane is corrected. So both geometrical detorsion and mechanical detorsion of the cylinder are working together. Untwisting the spine is done maintaining the curvatures in the sagittal plane. Indeed, the screw is not straight, but curved. However, curving the screwdriver is useless. The new solution is the moulding in frontal bending which respects lordosis and kyphosis and allows untwisting whilst retaining the curvatures in the sagittal plane. The spine in the sagittal plane is fixed as physiologically as possible. Only the frontal and horizontal planes are mobile (Figure 7).
5. The fifth concept according to Panjabi is the coupled motion behaviour of the spine. The moulding is 2D but the correction is 3D. The direction of rotation may differ depending on the incurvation of the spine in the sagittal plane. When there is a flat back, the initial scoliotic rotation may be increased by the correction in the frontal plane. Restitution of physiological curves in the sagittal plane seems to decrease the scoliosis rotation (Harrison Fryette’s laws)

Principle I: When the spine is in a neutral position, sidebending to one side will be accompanied by horizontal rotation to the opposite side.

Principle II: When the spine is in a flexed or extended position (non-neutral), sidebending to one side will be accompanied by rotation to the same side [24].

Although these laws have not been described in the context of scoliosis, we often see an accentuation of the rotation during pre-surgical bendings in supine position.

### Subjects

Since May 2013 all patients of JCdM at the ‘Clinique du Parc – Lyon’ were treated with the new Lyon brace (ARTbrace) instead of the classical EDF plaster cast. Our initial aim was to avoid the plaster cast, but very quickly, the ARTbrace appeared to be a much more effective solution compared to the former plaster casts and it was even better tolerated. So the whole treatment was continued with the same brace. In this prospective study of all patients of JCdM, the main group consisted of 225 patients with 304 curves from 20° to 55°. 245 primary curves with 26 double major curves and 59 secondary curves. Only patients with angulation exceeded 55° were excluded. Lumbar scoliosis continued to be treated with the short brace GTB [25]. The SRS/SOSORT criteria compliant group consisted of 64 patients with 84 curves.

All the data is recorded on Excel, and statistical analysis has been done with SPSS v20.

### Description of the brace

**Figure 8:**
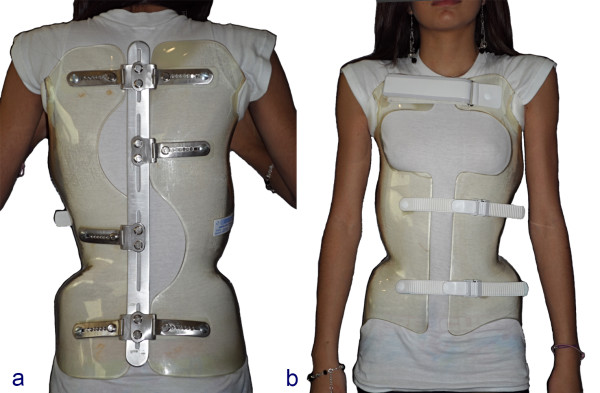
**ARTbrace: posterior (a) and anterior (b) view.**

**Figure 9:**
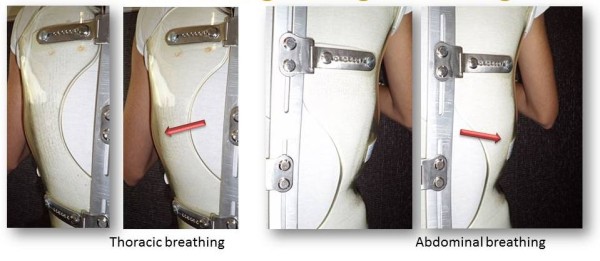
**Thoracic and lumbar expansion during breathing.**

ART is the Acronym for Asymmetrical, Rigid, Torsion brace. The name was created by Stefano Négrini, the inventor of the Sforzesco brace [26].Like the Sforzesco brace, the ARTbrace is constructed with 2 rigid asymmetrical lateral pieces of polycarbonate. They are connected posteriorly at the midline by a duraluminium bar like the historical Lyon brace. All metal parts are similar to those of the Lyon brace. Both anterior and lower ratcheting buckles are rigid, the upper third is Velcro (Figure 8).The brace is not in complete contact with the body: there is an expansion in the concavity which is there to allow room for the body’s expansion during inhalation (Figure 9).

### The new segmental CAD/CAM moulding

To obtain a torso column on the opposite side of the scoliosis, the superposition of three electronic instantaneous full 3D mouldings is necessary. These mouldings are made with the full 3D instantaneous raster stereography digitizer Orten. Markers are placed on the optical jersey:

On the front at the upper and lower part of the sternum and at the antero-superior iliac spine.

On the back on a point on each vertebral spinous process.

**Figure 10:**
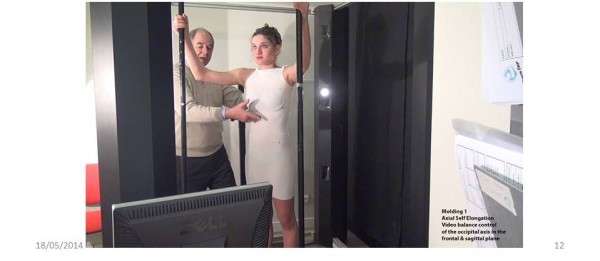
**Moulding 1 in axial self active elongation.**

**Figure 11:**
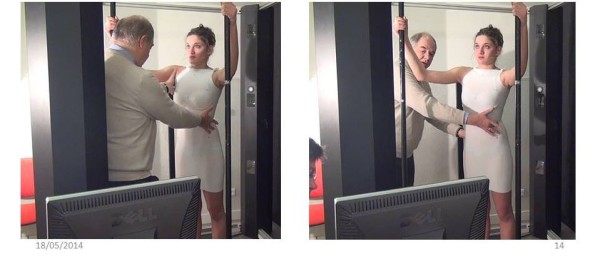
**Moulding 2 in lumbar shift and physiological lordosis.**

**Figure 12:**
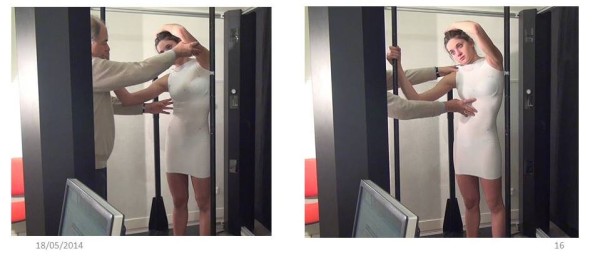
**Moulding 3 in thoracic shift and physiological kyphosis.**

A visually monitored control with a posterior and profile view is mandatory to obtain the ideal posture (Additional file 1).1. The First moulding is performed in self active axial elongation for the pelvis and the shoulders. Pelvic version and harmony of curvatures in the sagittal plane are monitored carefully, but without trying to correct them (Figure 10).2. The second moulding is performed in lumbar shift and physiological lordosis for the lumbar spine. On the concave side, the axillary-trochanter line is vertical (Figure 11).3. The third moulding is performed in thoracic shift and physiological kyphosis for the thoracic spine. On the concave side, the axillary-trochanter line is vertical. To improve the high thoracic shift, the hand is placed on the head which bows towards the concavity (Figure 12).

For a single thoracolumbar curve, both thoracic and lumbar shifts are made in the same direction.

### Modelling of the trunk shape with shapes overlay

**Figure 13:**
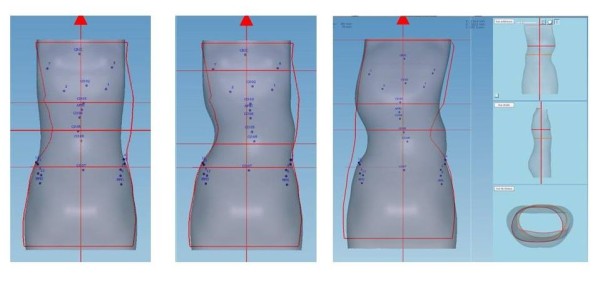
**Superposition in the frontal plane.**

**Figure 14:**
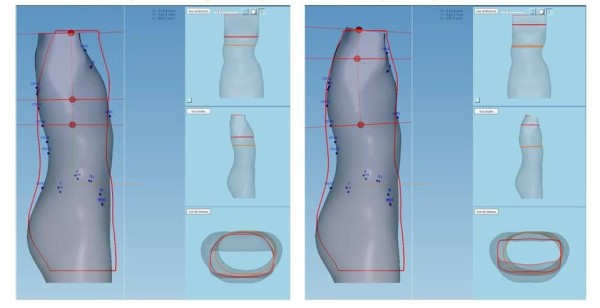
**Superposition in the sagittal plane.**

**Figure 15:**
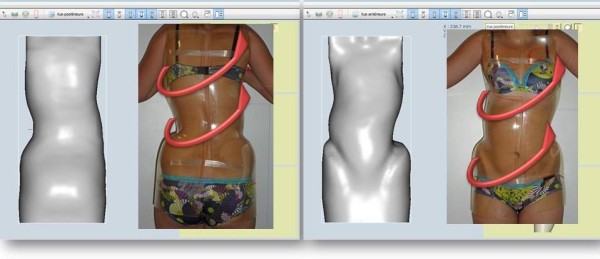
**Global helical detorsion after overlapping in the frontal and the sagittal plane.**

These modifications are made using the software OrtenShape.In the frontal plane moulding 2 is superimposed on moulding 1, then moulding 3 (Figure 13).Similarly in the sagittal plane, the second moulding is superposed on the first one, then on the third moulding (Figure 14).Changes are made at constant volume and detorsion which is a result of both corrections in the frontal plane and the sagittal plane (Figure 15).

### Specific design and curve pattern

A specific classification is not used, indeed most classifications were developed for surgery. For bracing, a specific classification was developed by Rigo for the specific needs of the RSC brace [27].

For the ARTbrace, the sagittal plane, pelvic tilt and axial balance are strictly controlled. The only modifications concern the frontal plane:

For a single thoracic curve, the second moulding is used only if the lordosis of the first moulding is incorrect and if this is the case we do not need the frontal shift.

For a single thoraco-lumbar curve, both thoracic and lumbar shifts will be made in the same direction.

For a double curve, the horizontal plane of overlay is at the level of the transitional vertebra, usually at the lumbosacral junction.

For a double thoracic curve, we give priority to the main rib hump, mainly the lower curve and in this case, the plastazote pad will be used to control the upper curve.

If the shoulders are unbalanced, it is also possible to make the upper end of the brace asymmetric at the axillary level like the historical Lyon brace.

No specific segmental derotation is required as the ARTbrace causes a global helical untwisting.

### 4D Global correction of the ARTbrace

**Figure 16:**
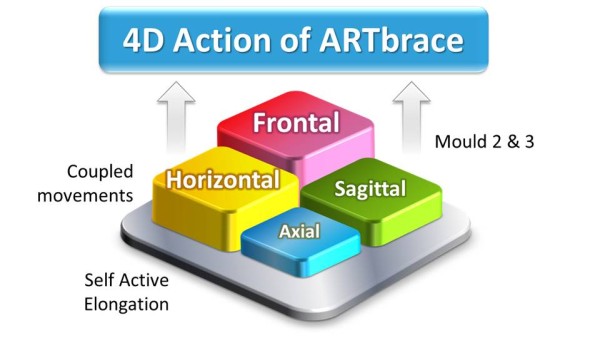
**4D Action of the ARTbrace.**

**Figure 17:**
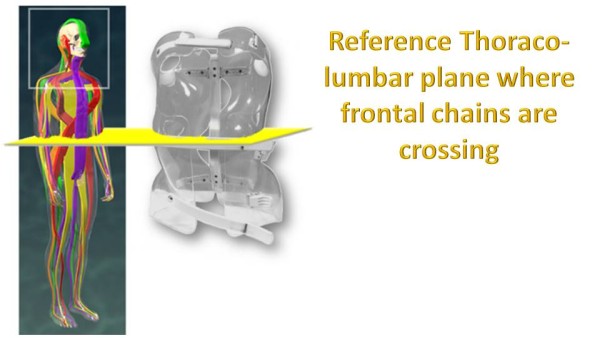
**Reference horizontal plane where muscular chains are crossing.**

**Figure 18:**
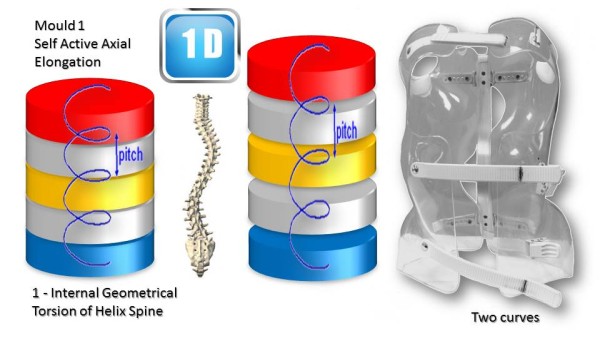
**First dimension; internal geometrical detorsion of helix.**

The mechanical action of the ARTbrace is carried out:

Along the vertical axis of the spineIn the three sagittal, frontal and horizontal planes of the spine (Figure 16).In ARTbrace, the reference plane is the horizontal plane at the thoracolumbar junction. The anterior and posterior muscle chains in the frontal plane intersect at this level. The middle brace closure with ratcheting buckle must be strict (Figure 17).The elongation along the axis of the spine is carried out during the first moulding. The spring effect moves the apical vertebrae near the spinal axis. This is the correction of the internal geometric vertebral torsion (Figure 18).

**Figure 19:**
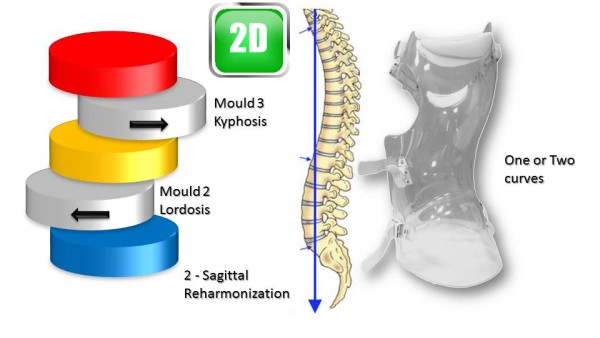
**Second dimension; restoration of physiological curvatures in the sagittal plane.**

**Figure 20:**
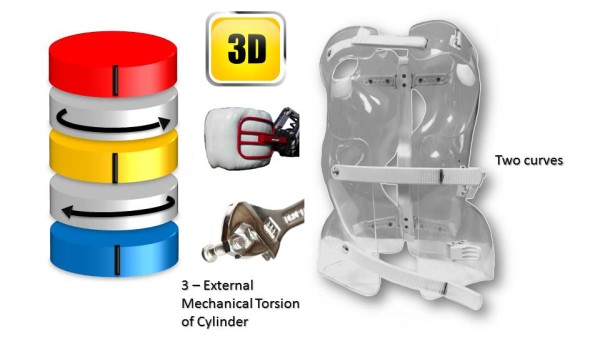
**Third dimension; external mechanical torsion of cylinder for a double curve.**

**Figure 21:**
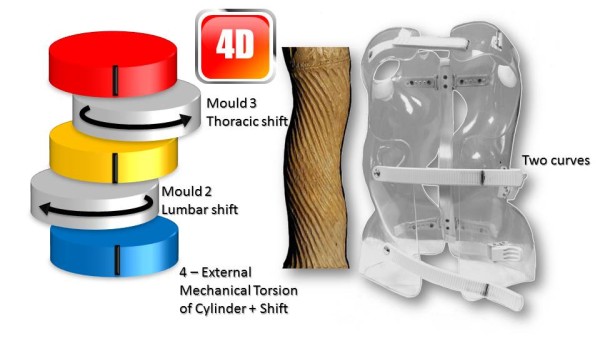
**Fourth dimension; Shift in the frontal plane.**

**Figure 22:**
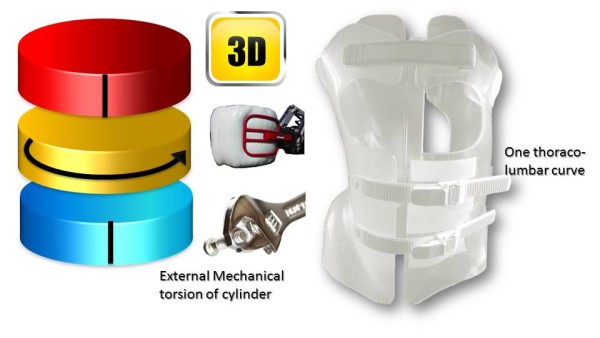
**Third dimension; external mechanical torsion of cylinder for a thoracolumbar curve.**

**Figure 23:**
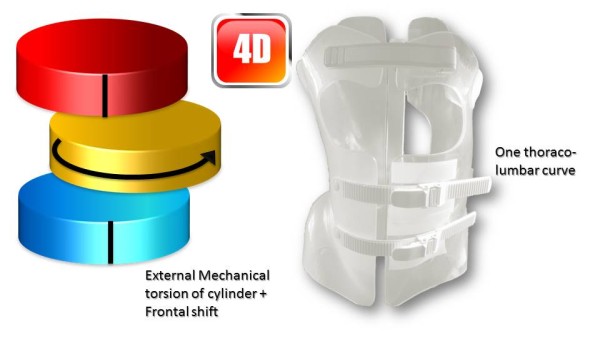
**Fourth dimension; frontal plane shift for a thoracolumbar curve.**

This classical elongation in braces such as the Milwaukee brace has the disadvantage of reducing the curvatures also in the sagittal plane.Segmental mouldings in the lumbar and thoracic areas overcome this disadvantage, and reproduce physiological curvatures in the fixed sagittal plane (Figure 19).The correction in the horizontal plane is on the whole external surface of the trunk. In the case of a double curvature, there is a first untwisting between the pelvis and the reference thoracolumbar plane, and a second untwisting between the reference plane and the shoulder girdle (Figure 20).The correction in the frontal plane is also exerted on the entire external surface of the trunk. It is the shift that is achieved with mouldings 2 and 3 which allows this correction. The translation is at the apical vertebra level and not below, as in the old Lyon brace (Figure 21).For a single thoracolumbar curve, it is the reference thoracolumbar plane which ensures derotation of the entire trunk, between both pelvic and scapular planes. The lever arm is more important and the curve is therefore better corrected (Figure 22).In the frontal plane, it is also the reference thoracolumbar plane that will translate between both scapular and pelvic girdles. Lumbar and thoracic shifts take place in the same direction (Figure 23).

The 4D global correction of ARTbrace occurs during the day and the movement is obtained by balancing among both frontal and horizontal anatomical planes. The inversion of the curvatures automatically creates an expansion in the concavity that allows the 4^th^ dynamical dimension, i.e. contact during movement and breathing.

### Practical issues

#### How to check the brace

Clinically, the height of the child in brace is measured, because the gain in height is an average of 1.58 cm due to the untwisting of the spine. This is an excellent clinical indicator of the effectiveness of the brace. In the sagittal plane, alignment of Tragus – Acromion - Trochanter - Ankles is checked.

Frontal and sagittal X rays are performed 3 to 4 days after fitting the brace with the ultra low dose EOS system which also allows a 3D reconstruction if necessary.

The metal bar must be vertical in the frontal plane and the C7 axis well balanced.

Adjusting the brace is made in the supine position. The middle ratcheting buckle is checked at the chondro-costal level. The tightening of the lower ratchet closure does not compress the abdomen, but stabilizes trochanters. Upper Velcro closure must be tight enough to prevent the tingling in the upper limbs.

**Figure 24:**
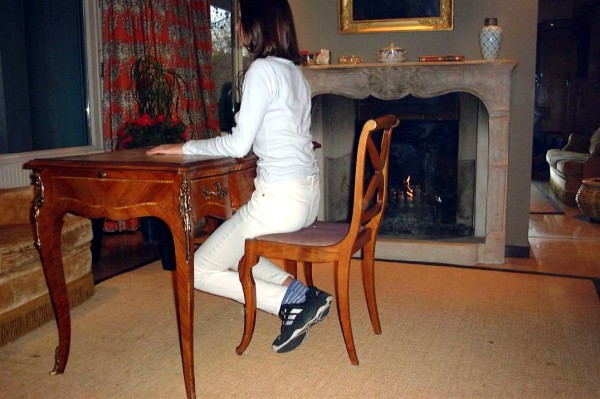
**Writing sitting posture.**

It is always possible to add on a plastazote pad inside the polycarbonate, but in practice this is an exception.Indications of the sitting posture are given with feet behind the chair, buttocks in front of the seat, polycarbonate touching the edge of the table and forearms on the table (Figure 24).

In the ARTbrace, the fixed point is the lower part of the thorax at the thoracolumbar junction. The dynamic movement of the posterior part of the spine is better in this posture. It is the fourth dimension of the brace. The child will relax in the listening posture on the back of the chair. Alternating these two extreme postures seems to be more dynamic.

#### Protocol and every day usage

All patients and parents give an informed consent and approval to use this new brace instead of the old plaster cast.

Similarly to the plaster cast, the total time is advised with weaning of a maximum of 10 minutes to allow for a shower.

Unlike clubfoot treated by serial casting according to the Ponseti method [28], there is little data in the literature regarding the time required to achieve a creep of the concavity in scoliosis. The Lyon experience is as follows: below 30° scoliosis, the total time is 1 month. The time required is two months for scoliosis between 30° and 39° and 4 months for scoliosis of more than 40° [3, 10, 11].

Indeed, continuous stretching for more than 3 weeks is necessary to permanently change the length of a ligament (creep), as for an ankle sprain. If the brace is removed for more than one hour, the viscoelastic structures return to their original length with only elasticity.

Physiotherapy is essential throughout the total time period; it is identical to that which was recommended with the plaster cast [29].

Sport is permitted with the brace and even recommended to better adjust the tension of the muscle chains. When the paraspinal musculature is active, it creates a pre-stressed beam along the spine which protects the vertebral body from collapsing [30].

## Results

All ARTbrace designs were based on the individual characteristics of the subjects’ scoliosis and segmental mouldings.

### Example

**Figure 25:**
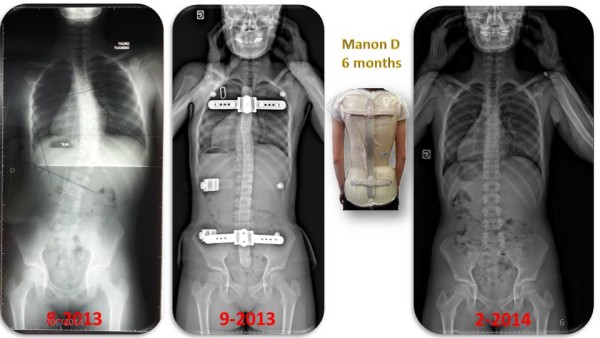
**First short time results of a single thoraco-lumbar curve.**

As an example we choose a scoliotic curve similar to the first subject of the RSC study [20] (Figure 25).

Manon, a girl of 12 years of age and Risser 0 was presenting an acute evolutive scoliosis with a progression of the Cobb angle from 15° to 39° in 4 months at the beginning of the treatment. At 6 months, the correction without the brace is highly effective.

### Frontal in-brace reduction

The main group has 225 patients with 304 curves from 20° to 55°. 245 primary curves with 26 double major curves and 59 secondary curves (Additional file 2).

The second group which meets SRS & SOSORT criteria has 64 patients with 84 curves:

from 25° to 40°

the age is 10 years or older when the brace is prescribed

Risser 0-2

no prior treatment

if female (61/64), either premenarchal or less than 1 year postmenarchal.

### Main group (n = 304)

The 158 thoracic curves are reduced by an average of 64%. The 146 lumbar curves are reduced by an average of 76%. For all curves the in-brace reduction is: 70% (Table 1).

**Table 1:** **In-brace correction of thoracic and lumbar curves of main group**

		Mean	Standard deviation	Percent change
Thoracic Cobb Angle (n = 158)	Before treatment	30°.39	7°.88	0.64
	3 days follow-up	11°.60	8°.28	
Lumbar Cobb Angle (n = 146)	Before treatment	28°.41	6°.64	0.76
	3 days follow-up	7°.51	8°.30	
			Average % change	0.70

The 245 primary curves are reduced on average by 72% and the 59 secondary curves of 60% (Table 2).

**Table 2:** **In-brace correction or primary and secondary curves of main group**

		Mean	Standard deviation	Percent change
Major Cobb Angle (n = 245)	Before treatment	30°.04	7°.67	0.72
	3 days follow-up	9°.29	9°.05	
Minor Cobb Angle (n = 59)	Before treatment	26°.97	5°.30	0.60
	3 days follow-up	10°.98	5°.69	
			Average % change	0.70

### SRS & SOSORT criteria (n = 84)

If we compile the same statistics for the 84 curves compliant with the SRS and SOSORT criteria, the percentage of in-brace correction is better: 66% for 41 thoracic curves, 82% for 43 lumbar curvatures; an overall correction of 75% (Table 3).

**Table 3:** **In-brace correction of thoracic and lumbar curves of SRS & SOSORT group**

		Mean	Standard deviation	Percent change
Thoracic Cobb Angle (n = 41)	Before treatment	30°.56	4°.918	0.66
	3 days follow-up	10°.46	6°.60	
Lumbar Cobb Angle (n = 43)	Before treatment	28°.51	4°.00	0.82
	3 days follow-up	5°.16	6°.74	
			Average % change	0.75

Similarly there is a 76% correction for the 72 primary curves and 70% for the 12 secondary curves (Table 4).

**Table 4:** **In-brace correction or primary and secondary curves of SRS & SOSORT group**

		Mean	Standard deviation	Percent change
Major Cobb Angle (n = 72)	Before treatment	30°.04	4°.56	0.76
	3 days follow-up	7°.64	7°.11	
Minor Cobb Angle (n = 12)	Before treatment	26°.33	3°.14	0.70
	3 days follow-up	8°.42	7°.66	
			Average % change	0.75

### Sagittal in-brace correction

The risk of overcorrection in the frontal plane is to accentuate the sagittal flat back.

The average angular thoracic kyphosis is 37° [31]. 94 patients had thoracic kyphosis under 30° before bracing. Data are summarized in (Table 5).

**Table 5:** **Mean and Standard deviation of sagittal in-brace correction (Cobb degrees)**

Paired samples statistics					
		Mean	N	Std. deviation	Std. error mean
Pair 1	Initial_kyphosis	19,41	94	6,964	,718
	Inbrace_kyphosis	28,59	94	5,701	,588

The Student *t*-test confirms that this correction is highly significant (Table 6).

**Table 6:** **Student** ***t*** **-test of Sagittal in-brace correction**

		Paired sample test					t	df	Sig. (2-tailed)
		Paired differences							
		Mean	Std. deviation	Std. error mean	95% confidence interval of the difference				
					Lower	Upper			
Pair 1	Initial_kyphosis	−9,170	6,465	,667	−10,494	−7,846	−13,752	93	,000
	Inbrace_kyphosis								

**Figure 26:**
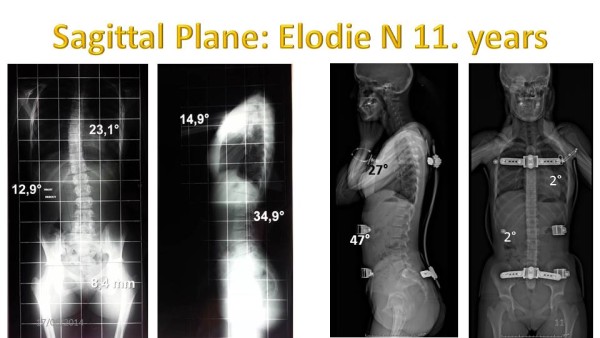
**Sagittal in-brace correction of Flat back.**

**Figure 27:**
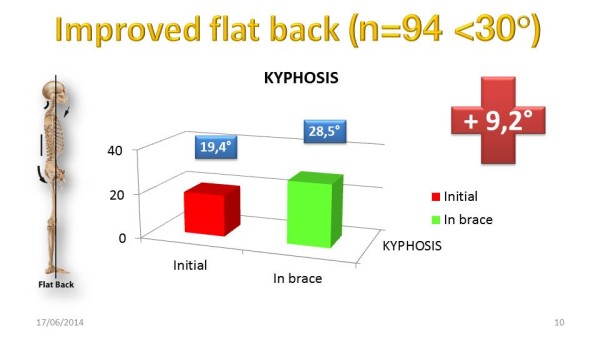
**Average Flat back improvement in ARTbrace.**

The nonparametric Wilcoxon test confirms that this correction is not related to chance (Table 7).In most cases, in-brace kyphosis is harmonious (Figure 26).The average improvement of kyphosis in ARTbrace is 9°.2 (Figure 27).

**Table 7:** **Wilcoxon of sagittal in-brace correction**

Hypothesis test summary				
	Null hypothesis	Test	Sig.	Decision
1	The median of differences between Initial kyphosis and in-brace Kyphosis equals 0	Related samples Wilcoxon Signed Rank Test	,000	Reject the null hypothesis.

Asymptomatic significances are displayed. The significance level is .05.

### Horizontal plane in-brace correction

**Figure 28:**
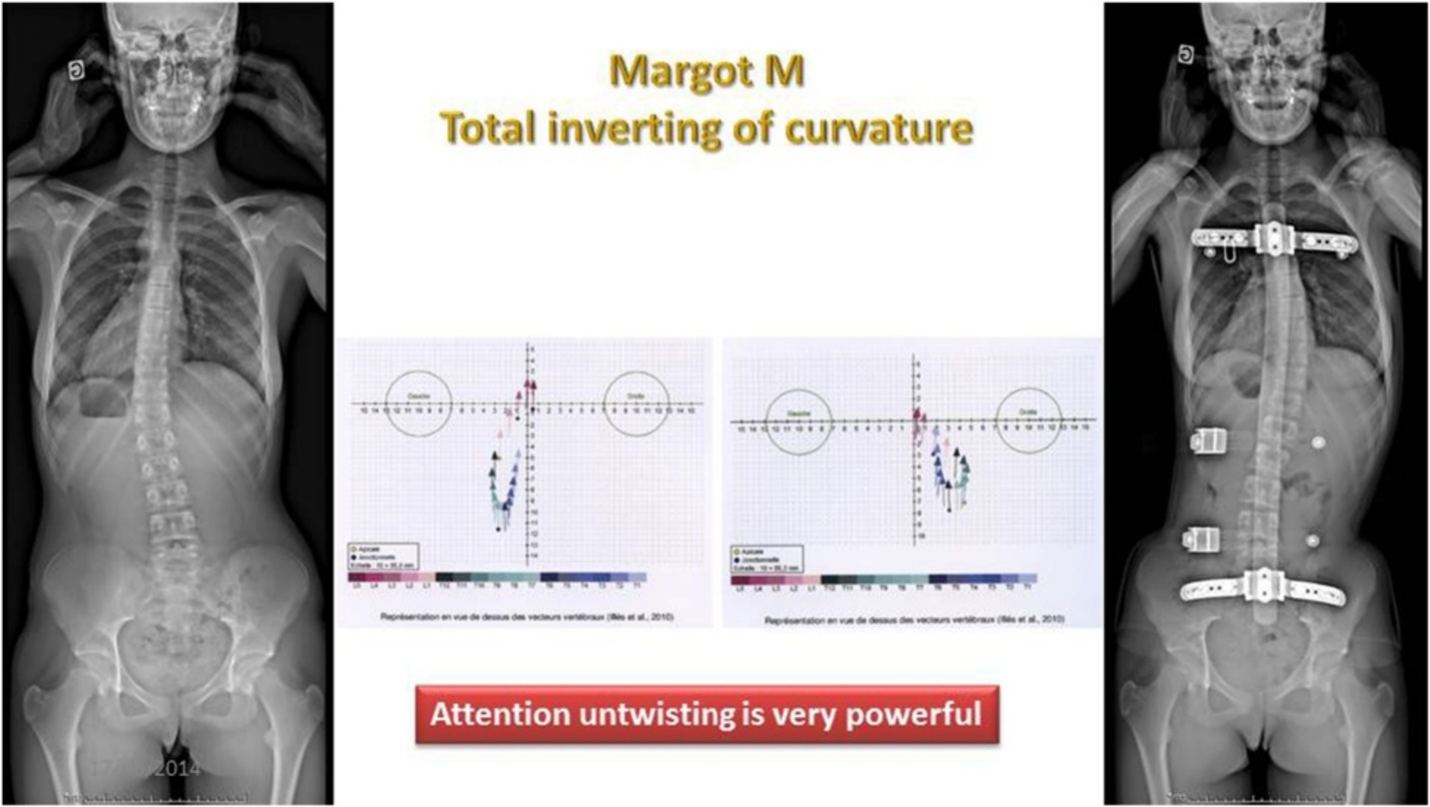
**Inversion of the curve without changing rotation.**

In a number of characteristic cases, the effect of the ARTbrace in the horizontal plane could be studied thanks to the EOS system. In most cases, the vertebral body is closer to the median vertical axis, but the rotation of each vertebra has changed very little. In the case of Margot who reversed her curvature in the ARTbrace, rotations remain identical (Figure 28).

## Discussion

The proposed concept should be verified with mathematical modelling and an advanced imaging technique (referred to the changes of bony geometry). All data are stored in ORTEN and constitute a database useful for further research.

The current clinical outcomes of ARTbrace look quite promising but are limited to short-term in this paper (3 days follow-up). Immediate in-brace angular reduction is not the final treatment outcome, 2 years after weaning the brace; but many authors use this value to assess the effectiveness of a brace and some authors are even using it as a predictive criterion [2, 32, 33]. Immediate in-brace reduction is related to the flexibility of scoliosis but also to the effectiveness of the brace.

**Figure 29:**
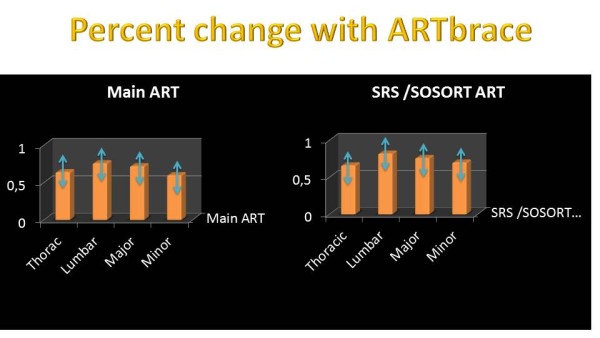
**Immediate in-brace Percent correction with ARTbrace.**

We can compare results in ARTbrace with the RSC [20]. The initial Cobb angle is 2° less in our series, but the correction is significantly different (Figure 29). The shapes of both curves are quite similar. The standard deviation, lower in our series, confirms the homogeneity of the prospective cohort.

Grivas [34] presents the in-brace correction of many European braces (Table 8).

**Table 8:** **Results of immediate in-brace correction of main European braces**

RCS [35]	all curves	0,42
Rigo System Chêneau	Primary curves	0.48
Scoliologic [36]	All curves	0,51
Chêneau light		
DDB [37]	all curves	0,495
Dynamic Derotation Brace		
PASB [38]	lumbar & Thor. Lumbar	0,499
Progressive Action Short Brace		

The results for North American braces are quite similar, with 0.5 for the Boston brace [33].

Castro [32], studying a prospective cohort of 41 AIS, concludes that the brace treatment is not recommended in patients whose correction is less than 0.20 in TLSO.

Appelgreen [39], in an article detailing measurement of the Cobb angle from the end vertebra in 121 AIS, concludes that an average in-brace correction of 0.30 gives hope a correction at the end of treatment.

Landauer [2], studying the predictive criteria of conservative treatment results in the first 6 months of treatment, wrote that compliant patients who have a high initial correction greater than 0.40 can expect a final reduction of about 7° and bad compliance is always associated with curve progression.

Wong [15], comparing the results of the electronic mould of 20 patients versus the traditional plaster moulding of 20 other patients, noted an improvement in the in-brace reduction from 0.32 to 0.42 in support of CAD/CAM moulding.

Bullmann [40], presenting the prospective results of 52 patients treated with the Chêneau-Toulouse-Münster brace with curves between 25° and 40°, estimated the in-brace correction at 0.43. There was a positive correlation between flexibility and Cobb angle correction during brace treatment and a negative correlation between Cobb angle correction during brace treatment and curve progression.

In the sagittal plane, the correction obtained in the flat back is unique today. Indeed, most authors consider that the correction in the frontal plane is related to axial stretching accentuating the flat back [20]. With the ARTbrace there is certainly an extension, but the main part of the correction is made by unscrewing or untwisting the spine with translation of the vertebral bodies near the midline.

After more than one year using the ARTbrace, we can summarize some improvements (in alphabetical order):

**4D action**: hypercorrection action in the frontal and horizontal planes during breathing and motion.

**Aesthetics**: the brace is transparent, almost invisible under clothing. However, the asymmetrical ARTbrace is less aesthetic than the symmetrical Sforzesco brace.

**Economy**: no more plaster casts, no more hospitalisation, and the life-span of the brace is greater than that of the plaster cast.

**Efficiency**: the brace is adjustable in the frontal plane; an additional correction by internal pad is easy.

**Hygiene**: a daily 15-minute shower is possible.

**Insulation**: the polycarbonate is more insulating than the glass and there is no need for perforation.

**Lightness**: it is the end of 5-7 kg plaster casts, and the ARTbrace is 25% lighter than the historical Lyon brace.

**Originality**: this is the first untwisting brace of the whole spine in three planes of space.

**Simplicity**: anyone can make a frontal bending with lordosis or kyphosis; no major correction of the positive mould is necessary, like the Chêneau brace.

**Tolerance**: polycarbonate is biologically well tolerated.

**Universality**: it is possible to correct hyperkyphosis like hypokyphosis.

## Conclusions

This first paper is an introduction with very short results and does not prejudge the final outcome, but during the last 50 years, the immediate in-brace reducibility of scoliosis remained around 0.50 and progress focused on aesthetics and tolerance. Thanks to advances in computer graphic technology this correction exceeds for the first time 0.70 with the ARTbrace.

This correction requires no more significant alteration of the positive mould, but the superposition of three segmental CAD/CAM in a simple and strictly defined posture.

Improving the flat back in the sagittal plane has never been described with scoliosis braces used to date.

Lyon brace management and protocol are not modified by the use of the ARTbrace and a priori the final results of the treatment cannot be worse than the historic Lyon brace.

While the ARTbrace could be defined as a modified or “new” Lyon brace, the new concepts and first results prove that it can completely replace the casting and old Lyon brace process; it really deserves to be recognized, as its unique design has surpassed its predecessor and former protocol.

Further results will be published separately in due course:

comparing the tolerance with plaster cast using the questionnaire BRQ,

the aesthetic results,

the first results at 6 months and one year compared with the historical Lyon brace in a matched case-control study.

## Electronic supplementary material

Additional file 1: **de Mauroy’s segmental moulding for ARTbrace.** (MP4 15 MB)

Additional file 2: **Database of 225 first consecutive patients treated with ARTbrace.** (XLSX 36 KB)

## Abbreviations

ARTbrace: Acronym for Asymmetrical Rigid Torsion brace created by Stéfano Négrini
RSC: Rigo System Chêneau
BrQ: Acronym for Brace Questionnaire from Vasiliadis & Grivas validated in France by Julie Deceuninck
Cad/Cam: Acronym for computer-aided design/computer-aided manufacturing
EOS: Radiological device using ultra low dose irradiation
BRAIST: Acronym for Bracing Scoliosis in Adolescent idiopathic Scoliosis trial
AIS: Acronym for Adolescent Idiopathic Scoliosis
EDF: Acronym for Elongation Derotation Flexion (Cotrel’s frame).
